# Evaluation of Group Therapy Intervention for Anxiety and Depression in the Postnatal Period

**DOI:** 10.1007/s10995-025-04076-9

**Published:** 2025-03-26

**Authors:** Jessica Appleton, Cathrine Fowler, Lisiane Latouche, Jenny Smit, Margaret Booker, Greg Fairbrother

**Affiliations:** 1 Tresillian Family Care Centres, McKenzie Street, Belmore, Sydney, NSW 2192 Australia; 2 School of Nursing and Midwifery, Faculty of Health, University of Technology Sydney, 15 Broadway, Ultimo, NSW 2007 Australia; 3 Sydney Local Health District, Camperdown, NSW 2050 Australia

**Keywords:** Postnatal depression, Postnatal anxiety, Mothers, Group psychotherapy, EPDS

## Abstract

**Objectives:**

Effective interventions for the prevention and treatment of postnatal depression and anxiety are important for family functioning and infant development. Group psychotherapy is one such intervention used in the postnatal period. This evaluation tests the efficacy of an eight-week group psychotherapy on participants’ symptoms of postnatal depression and anxiety. The study also reports on the addition of a sequential attachment-based parenting program on participants’ symptoms of depression and anxiety.

**Methods:**

The study setting was a child and family health service in New South Wales, Australia. 141 women participating in a group psychotherapy program consented to participate in the study. A pre-test post-test design with four week follow up was used. Depression was measured by the Edinburgh Postnatal Depression Scale (EPDS) and anxiety with the anxiety subscale (EPDS-3a). Repeated measures analysis of variance and multiple factorial mixed analysis of variance were conducted.

**Results:**

The mean EPDS score reduced significantly from 14.11 pre-intervention to 9.99 (*p* < .001). The mean EPDS-3a score reduced significantly from 5.68 pre-intervention to 4.37 (*p* < .001). These improvements were still present four weeks after completion of the program at a ‘group reunion’ session. The addition of a sequential attachment-based parenting program was not shown to be effective in further reducing symptoms of depression or anxiety (*p* =.183).

**Conclusions for Practice:**

These findings support the efficacy of group psychotherapy interventions for reducing both depression and anxiety symptoms in the postnatal period. This intervention demonstrated lower depression symptoms in the post test compared to the pre-test periods for women with and without previous history of mental illness and those with varied baseline depression symptoms.

**Supplementary Information:**

The online version contains supplementary material available at 10.1007/s10995-025-04076-9.

## Significance

What is already known on this subject?

What this study adds?

Group psychotherapy can be an effective intervention for women experiencing both postnatal depression and anxiety.

The eight-week psychotherapy group was effective in reducing symptoms of postnatal depression and anxiety for women with and without previous history of mental illness and those with and without baseline depression symptoms within the clinical range. The addition of a sequential attachment-based parenting program was not shown to be effective in further reducing symptoms of depression or anxiety.

**Supplementary Information:**

The online version contains supplementary material available at 10.1007/s10995-025-04076-9.

## Introduction

Many women are at risk of experiencing symptoms of depression and anxiety in the postnatal period (Dennis et al., 2017; Shorey et al., 2018). Whilst experiencing a range of emotions during the postnatal time is considered normal, for some women, emotion-related symptoms can be more intrusive and debilitating and can lead to sub-optimal coping (e.g. emotional withdrawal). Such symptoms may place a woman into the range where a formal diagnosis of a major depressive disorder (American Psychiatric Association (APA), 2013) and/or a generalised or social anxiety disorder (APA, 2013) may be made. These disorders are commonly known as postnatal depression (PND) and postnatal anxiety (PNA).

It is estimated worldwide that 16–20% of women experience PND (Shorey et al., 2018) and between 15 and 18% experience PNA symptoms (Dennis et al., 2017) in the *first year* after birth. Local data of PND within the *first 6-week* post-partum suggest a prevalence of 3.3–6.2% (Eastwood et al., 2011; Ogbo et al., 2018). Whereas Australia-wide population prevalence estimates of women experiencing either PND or PNA in the *first-year* post-partum is 20% (PwC Consulting, 2019). Indicating prevention, screening, diagnosis and treatment options should be available throughout the first year after birth. PND and PNA are associated with lower quality of life, greater perceived stress, lower social functioning, more relationship difficulties, and negative impacts on the development of infant-mother attachment (Slomian et al., 2019). There are also short- and long-term implications for infants’ emotional, social, and behavioural development (Slomian et al., 2019). Therefore, effective prevention, screening, diagnosis and treatment of both PND and PNA are important for the short- and long-term health of both women and infants. Psychosocial risk factors that place women at a higher risk of experiencing PND and PNA have been identified. These include lower socio-economic status and history of mental illness (McCarter-Spaulding & Shea, 2016).

There is evidence that group psychotherapy is an effective approach for women experiencing PND and PNA. Two previous systematic reviews with meta-analyses demonstrated cognitive behaviour therapy (CBT) delivered both in group and via other modes (e.g. individual in person treatment or virtual treatment) is effective in reducing depressive symptoms (Li et al., 2022; Scope et al., 2013) and anxiety symptoms (Li et al., 2022). Similarly, quasi-experimental (pre- post- design) studies have shown group CBT as effective in reducing both depression and anxiety symptoms (Furer et al., 2021; Green et al., 2020; Simhi et al., 2021). Group psychotherapy based on other psychological strategies have also been shown to be effective, for example group interpersonal therapy (Mulcahy et al., 2010; Reay et al., 2012), mindfulness based cognitive therapy (Li et al., 2022) and acceptance and commitment therapy (Waters et al., 2020). The combination of CBT and mindfulness has been identified as particularly effective for PND (Li et al., 2022). Existing qualitative research suggests that group therapy is acceptable for most women (Scope et al., 2012). It remains the case though that much of the research has been conducted among women with diagnosed PND. There is limited evidence on the efficacy of group psychotherapy among those with sub-clinical symptomatology of PND and PNA.

There is emerging evidence that attachment-based parenting programs can have a positive impact on maternal psychopathology (Huber et al., 2016; Kim et al., 2018; Maupin et al., 2017; Maxwell et al., 2021). It is well known that there is an interaction between depression and mother-child bonding and/or relationship (Slomian et al., 2019), with mothers experiencing depression also experiencing more difficulties in their relationship with their infant (Slomian et al., 2019). Attachment-based parenting program can have positive impact on both maternal-child relationships and maternal depression (Maxwell et al., 2021). Previous case and pre- post- intervention studies of the attachment-based parenting program, Circle of Security parenting groups (COS-P), have identified improvements in participant symptoms of depression (Huber et al., 2016; Kim et al., 2018; Maupin et al., 2017). However, the mechanism of action and direction of effect are still unclear (Huber et al., 2016; Maxwell et al., 2021). The primary objective of this study was to evaluate the Tresillian group psychotherapy program to identify effects on participants’ symptoms of postnatal depression and anxiety. A secondary aim was to explore any differences found in relation to baseline characteristics including known risk factors such as previous history of mental illness, socioeconomic status, and the existence of clinical or sub-clinical symptoms of depression before commencing the program. A third objective was to explore if combining the Tresillian group psychotherapy with a follow-on attachment-based parenting program would have any impact on symptoms of depression and anxiety.

## Methods

### Participants

Between 2017 and 2019 women who were participants in the Tresillian group psychotherapy program, ‘Postnatal Depression & Anxiety’ group, were invited to participate in the evaluation. This program is advertised as a therapeutic group for the treatment of perinatal depression and anxiety and is provided free to any eligible women in the community through a practitioner-referral. The practitioner referral can be internally from practitioners within the service the group is run or external in other primary health care services (e.g. specialised child and family health nurses or General Practitioners [generalist primary health care medical doctor]). For either referral pathway women have an initial telephone screening with a clinician who will assess eligibility for inclusion in the program. The eligibility criteria include being within 24 months postpartum and experiencing self-reported postnatal stress, depression and/or anxiety of a non-acute nature, notably while the clinician discusses how the participant is feeling at this initial telephone meeting there is no specific diagnostic or screening threshold for participation in the program. All women participating in the program at the evaluation sites were eligible to be included in the evaluation. Due to this open referral pathway and no diagnostic or screening threshold there was heterogeneity in the characteristics of the women in the program. They may have had a history of mental illness, be first- or subsequent-time mothers and, as measured before the first session, may or may not report symptoms of PND and/or PNA that would be described as ‘clinical’ (i.e. an Edinburgh Postnatal Depression Scale (EPDS) > 13). The group include mothers of infants and toddlers up to 24 months in age.

### The Program

Tresillian is a tertiary referral child and family health service in NSW Australia. The group psychotherapy program is conducted in-person at multiple metropolitan sites. Based on practical considerations of data collection of an existing program this evaluation was limited to two sites. Site 1 is located in an area of relative socioeconomic disadvantage based on the Australian Bureau of Statistics Index of Relative Socio-economic Advantage and Disadvantage for geographically-defined areas (Australian Bureau of Statistics, 2018). The group program consisted of eight weekly two hourly sessions (16 h) with up to 12 women per group and two trained facilitators. In addition to these core sessions there is a follow up session ‘group reunion’ session which is held four weeks after the final core sessions and a single psycho-education session for the women’s partner is held during an evening around week five or six of the core sessions.

The core sessions included the following topics:

1. Overview of postnatal depression/anxiety symptoms and treatment options.
2. Managing moods: CBT, mindfulness and self-compassion.
3. Effectively communicating one’s needs.
4. Journey of the self: identity as a mother.
5. Life Journey: a narrative approach.
6. Re-connecting to my child’s father.
7. Parent infant relationship.
8. Change is recovery.

The two trained facilitators can be a registered nurse with child and family health qualification and either a registered social worker or psychologist. At the two sites included in this evaluation the facilitators were a social worker and a specialised child and family health registered nurse. The ‘Tresillian PNDA group’ has a standardised manual and training for facilitators involves a 2-day (16 h) training course run by Tresillian.

The PNDA group therapy takes a strengths-based approach, is informed by attachment theory (Bowlby, 1998) and is based on a range of cognitive therapies, including mindfulness-based cognitive therapy (Williams et al., 2007), dialectical behaviour therapy (Dimeff & Koerner, 2007) and acceptance and commitment therapy (Zettle, 2007). Group therapy designed with a mix of theoretical approaches is not uncommon (Gillis & Parish, 2019). A recent integrative review identified a conceptual model for understanding the process and outcomes of group therapy for PND (Gillis & Parish, 2019). They identified a key theme for women attending this type of group was the group environment itself; where a space of shared experience and understanding amongst the group was valued (Gillis & Parish, 2019). The theoretical understanding of group therapy incorporated into the ‘Tresillian PNDA group’ approach is that interpersonal interactions are key and quality and consistent participation is a critical factor for successful outcomes in group therapy (Yalom & Leszcz, 2005). Given this, the facilitator role is to build a therapeutic alliance and facilitate a safe space and value group members (Scope et al., 2012). They do this by keeping the whole group in mind, being mindful of affect and fostering ongoing participation by for example, promoting the use of personal language, expressing feelings and attentive listening. The facilitator role is crucial in guiding the group and best practice guidelines highlight the importance of interventions led by trained professionals (Highet & the Expert Working Group and Expert Subcommittees 2023).

Tresillian also facilitate Circle of Security parenting groups (COS-P) (Powell et al., 2013). COS-P is a widely accepted attachment-based parenting program which educates parents about attachment and develops parent’s skills for observing and identifying their child’s cues and skills in responding in a sensitive and supportive way (Kohlhoff et al., 2016; Powell et al., 2013). In addition to the eight-week Tresillian PNDA group, there was also a sequential PNDA with COS-P group. These groups, after completing the eight weeks, then ran the COS-P program over four consecutive weeks, with four-hour sessions each week (16 h).

### Study Design

A pre-test post-test design was employed utilising the EPDS to measure depression and anxiety symptomatology. Baseline measures were taken at the start of the first session. Post*-*tests were administered post-program (week 8), at the end of the final session and 4-week follow up (week 12), which was at the ‘group reunion’ session. Measures were self-administered on paper collected as part of the usual care and transcribed into a secure database.

### Measures

The Edinburgh Postnatal Depression Scale (EPDS; Cox et al., 1987), a 10-item self-report questionnaire was used to measure depressive symptoms. This measure has been shown to identify postnatal depression and anxiety symptomatology and is a commonly used tool to measure PND symptoms for group-based intervention (Gillis & Parish, 2019; Scope et al., 2013). Several studies have shown that the EPDS-3a, a three-item subscale of the EPDS, provides a specific measure of anxiety symptoms (Matthey et al., 2013; Phillips et al., 2009), this was therefore used to measure anxiety symptoms.

### Statistical Analysis

Descriptive statistics were used to describe the sample. The characteristics of participants who were included in the final analysis were compared to those who were excluded (for example, due to loss to follow up) using the Pearsons chi-square test. Repeated measures analysis of variance (ANOVA) was conducted to compare EPDS and EPDS-3a outcome across the three time points. Multiple factorial mixed ANOVAs were conducted which included the three time points, using EPDS and EPDS-3a measures separately as dependant variables, and the following independent variables: Group ID (11 groups included in analysis); PNDA group versus PNDA + Cos group; clinical versus sub-clinical EPDS score at baseline; socio-economically defined group location (Site A and Site B); level of education and history of mental illness. Missing data were examined. For the EPDS and EPDS-3a, data those data missing at random were imputed with the whole group mean. An available-case sub-group analysis was conducted (appendix 1). All statistical analyses used two tailed significance tests with an alpha level set at 0.05 and conducted in SPSS version 28 (IBM Corp., Released 2021).

### Ethics

Participant information statements were given to potential participant’s at the first session, where they had an opportunity to ask questions. Consent for inclusion in the study was gained prior to data collection. The study was approved by the Sydney Local Health District Human Research Ethics Committee (X16-0181, LNR/16/RPAH/222).

## Results

**Fig. 1:**
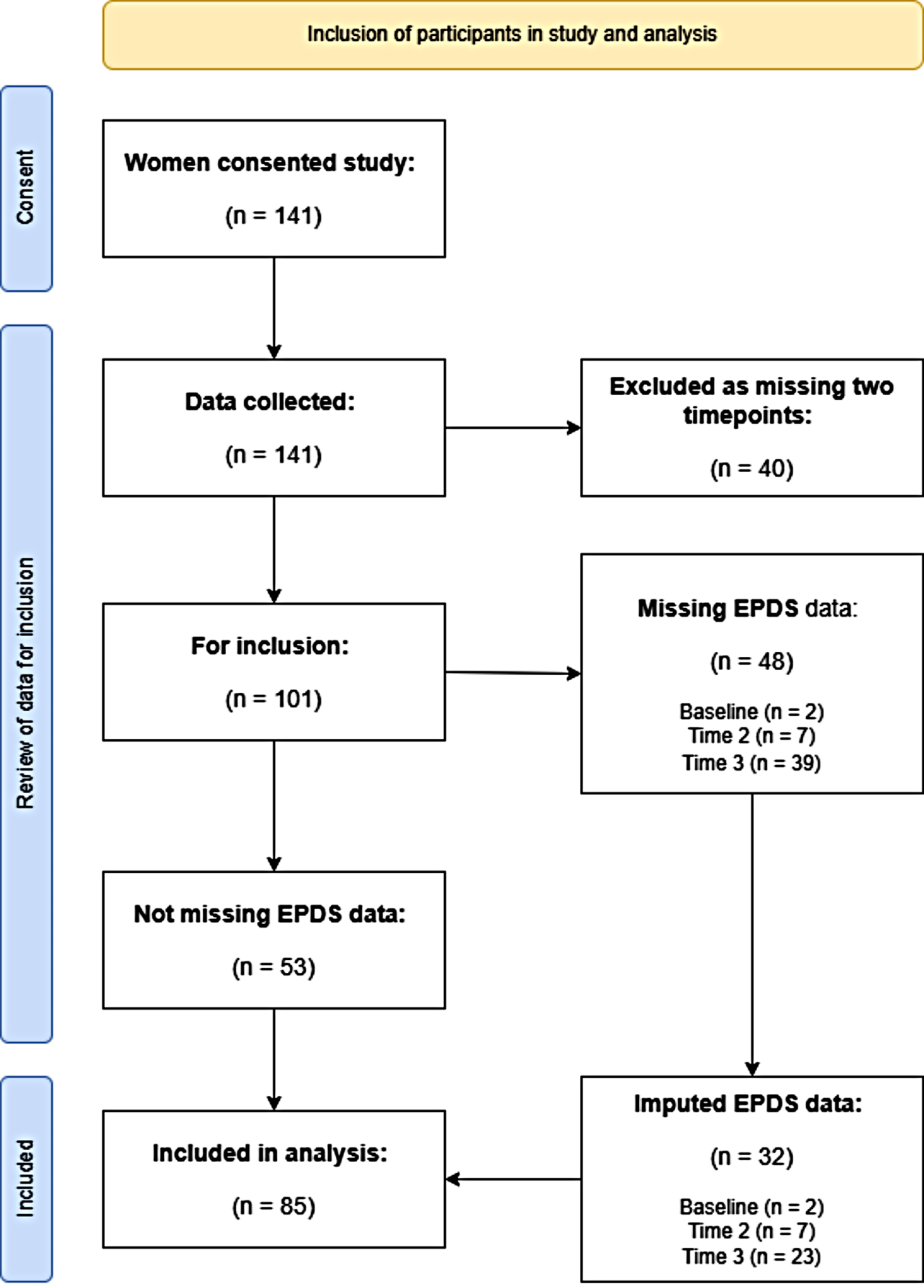
Participants included in analysis

A total of 141 women consented to enrol in the study. Forty (*n* = 40) were excluded from the analysis due to missing data collect at two timepoints (e.g. loss to follow-up). The final sample *n* = 101 was examined for missing data. A total of *n* = 48 participants were missing EPDS data. For two of the treatment groups (*n* = 16) all EPDS data were missing (missing not at random). For the remaining missing EPDS data, EPDS and EPDS-3a scores were imputed with the mean (see Fig. 1). There was no difference between those included and excluded in the analysis by EPDS baseline score, age, education, or history of mental illness (see Appendix 1). There was a difference by group and site, with more participants from site 1 and PND + Cos groups excluded from the analysis (see Appendix 1).

Among those included in the analysis, the majority had EPDS scores within the clinical range (65.9%) and had a history of mental illness (55.3%). The majority were university educated (70.6%), 30–39 years old (78.8%) and had only one child (63.5%) aged under 12 months (Table 1).

**Table 1:** Sample characteristics

	*n* (%)	Missing, *n*(%)
**EPDS baseline score**		*n* = 2 (2.4%)
Clinical	56 (65.9%)	
Sub-clinical	27 (31.8%)	
**Group**		*n* = 0
PND	65 (76.5%)	
PND + COS-P	20 (23.5%)	
**Site**		*n* = 0
Site 1 lower socioeconomic area^	35(41.2%)	
Site 2 higher socioeconomic area	50 (58.8%)	
**Mother age**		*n* = 8 (9.4%)
20–24	1 (1.2%)	
25–29	4 (4.7%)	
30–34	25 (29.4%)	
35–39	42 (49.4%)	
40–44	4 (4.7%)	
44+	1 (1.2%)	
**Mother Highest education**		*n* = 12 (14.1%)
Secondary School	2 (2.4%)	
Higher Secondary School	1 (1.2%)	
Certificate or Diploma	10 (11.8%)	
Bachelor’s Degree	34 (40%)	
Post-Graduate Certificate or Diploma	6 (7.1%)	
Masters or Doctoral degree	20 (23.5%)	
**Number of Children**		*n* = 12 (14.1%)
1	54 (63.5%)	
2	15 (17.6%)	
3	3 (3.5%)	
4	1 (1.2%)	
**History of Mental illness**		*n* = 17 (20%)
Yes (and not sure)	47 (55.3%)	
No	21 (24.7%)	
**Youngest Child age**		*n* = 5 (5.9%)
Mean (SD); Range	6.4 months (SD 3.5), 1.2–17 months	
**Number of sessions attended**		*n* = 31 (36.5%)
	Mean: 7.2, Range 4–8 sessions	

^Based on the Australian Bureau of statistics Index of Relative Socio-economic Advantage and Disadvantage (IRSAD) Socio-economic indexes for areas (Australian Bureau of Statistics, 2018)

Repeated measures ANOVA identified significant improvement in EPDS (*p* < .001) and EPDS-3a (*p* < .001) between baseline and at the end of the last PNDA group session (Table 2). There was no further change in symptoms of depression and anxiety between the end of the last PNDA group session (week 8) and the 4-week follow up (week 12), with no significant difference in scores for both EPDS (*p* =.631) and EPDS-3a (*p* =.651, Table 2).

**Table 2:** Repeated measures ANOVA

	**EPDS score (95% CI)**				**Overall statistic**
	**Baseline**	**Post-group (week 8)**		**4-week follow up (week 12)**	
All (*n* = 85)	14.11 (13.24–14.99)	9.99 (9.1–10.88)		9.77 (8.91–10.64)	*F* = 44.08 (1.93, 161.67), *p* < .001
*Contrast statistic*	*F* = 54.84(1, 84), *p* < .001		*F* = 0.23(1, 84), *p* =.631		
	**EPDS-3a score (95% CI)**				
	**Baseline**	**Post-group (week 8)**		**4-week follow up (week 12)**	
All (*n* = 85)	5.68 (5.31–6.06)	4.46 (4.09–4.82)		4.37 (4.02–4.73)	*F* = 27.98(2, 168), *p* < .001
*Contrast statistic*	*F* = 39.52(1, 84), *p* < .001		*F* = 0.21(1, 84), *p* =.651		

Factorial mixed ANOVA identified that some variables had significant impact on score for EPDS and EPDS-3a over the three time points. Specifically, group membership and EPDS score showed a significant interaction effect (*p* =.007) indicating the group change over time was not uniform between each of the 11 groups (see Table 3 & Appendix 1). This same effect was not detected for EPDS-3a (see Table 4). A significant main effect difference was located between the PNDA and PNDA + COS-P groups (EPDS, *p* =.029; EPDS-3a, *p* =.039). This identified a significant difference in EPDS and EPDS-3a between these groups at the week eight time point (*p* =.012). However, the interaction effect between these groups was not significant (*p* =.370), indicating that the degree of change over time for both groups was not different (Tables 3 and 4).

**Table 3:** Factorial mixed ANOVA EPDS

Independent variable	Main effects		Interaction effects		Between group effects by time point			
	F-statistic	*p*-value	F-statistic	*p*-value	Reference group	Pre	Post	Reunion
Group (*n* = 85)	F(10,74) = 0.54	*p* =.849	F(20,148) = 2.08	*p* =.007	See appendix			
Group PND only or PND + CoS (*n* = 85)	F(1,83) = 4.96	*p* =.029	F(1.95,161.48) = 1.0	*p* =.370	PND + CoS	− 0.96 (-3.02 – 1.10) *p* =.355	-2.63 (-4.67 - − 0.56) *p* =.012	-1.37 (-3.39 – 0.66) *p* =.183
Clinical or sub-clinical baseline EPDS (*n* = 83)	F(1,81) = 26.21	*p* < .001	F(2, 162) = 17.03	*p* < .001	Clinical	-6.6 (-7.85 - -5.35) *p* < .001	-1.25 (-3.17 − 0.68) *p* =.201	-1.55(-3.39 − 0.29) *p* =.097
Site (*n* = 85)	F(1,83) = 1.45	*p* =.232	F(1.94,161.39) = 0.28	*p* =.750				
Education (*n* = 73)	F(1,71) = 0.92	*p* =.340	F(1.99, 141.6) = 0.77	*p* =.465				
Hx Mental illness (*n* = 68)	F(1,66) = 6.73	*p* =.012	F(2, 132) = 0.12	*p =*.885	Yes	-2.19 (-4.35 - − 0.03) *p* =.047	-2.33 (-4.57 - − 0.09) *p* =.042	-1.72 (-3.87 − 0.44) *p* =.116

**Table 4:** Factorial mixed ANOVA EPDS-3a

Independent variable	Main effects		Interaction effects		Between group effects by time point			
	F-statistic	*p*-value	F-statistic	*p*-value	Reference group	Pre	Post	Reunion
Group (*n* = 85)	F(10,47) = 1.84	*p* =.069	F(20,148) = 1.26	*p* =.213				
Group PND only or PND + CoS (*n* = 85)	F(1,83) = 4.38	*p* =.039	F(2,166) = 0.829,	*p* =.438	PND + CoS	-0.48 (-1.37 − 0.41) *p* =.286	-1.04 (-1.88 - − 0.20) *p* =.016	− 0.58 (-1.41 − 0.24) *p* =.164
Clinical or sub-clinical baseline EPDS (*n* = 83)	F(1,81) = 12.26	*p* =.001	F(2, 162) = 9.87	*p* < .001	Clinical	-2.06 (-2.76 - -1.37) *p* < .001	− 0.55 (-1.33 − 0.23) *p* =.167	− 0.49 (-1.28 − 0.27) *p* =.203
Site (*n* = 85)	F(1,83) = 2.06	*p* =.155	F(2,166) = 0.52	*p* =.595				
Education (*n* = 73)	F(1,71) = 1.83	*p* =.181	F(2,142) = 0.16	*p* =.857				
Hx Mental illness (*n* = 68)	F(1,66) = 7.83	*p* =.007	F (2,132) = 0.06	*p* =.940	Yes	-1.06 (-2.01 - − 0.12) *p* =.028	− 0.9 (-1.82 − 0.019) *p* =.055	-1.01 (-1.83 - − 0.195) *p* =.016

There was also a significant main effects difference between those with and without a history of mental illness (EPDS, *p* =.012; EPDS-3a, *p* =.007). Those with a history of mental illness had significantly higher EPDS scores at baseline (*p* =.047) and week eight time point (*p* =.042) compared to those without a history of mental illness. Those with a history of mental illness had significantly higher EPDS-3a scores at baseline (*p* =.028) and week 12 time point (*p* =.016), compared to those without a history of mental illness. However, again, there was no significant interaction effect in each case (EPDS, *p* =.885; EPDS-3a, *p* =.940), indicating that the degree of change over time for those with and without a history of mental illness was similar (Tables 3 and 4).

There were significant main effects between those with clinical or sub-clinical baseline EPDS scores (EPDS, *p* < .001; EPDS-3a, *p* < .001). As expected, at baseline the sub-clinical group had significantly lower EPDS and EPDS-3a scores. There was also a significant interaction effect between clinical or sub-clinical baseline EPDS and both depression and anxiety symptoms over time (EPDS, *p* < .001; EPDS-3a, *p* < .001, Tables 3 and 4). This finding indicates that those with ‘sub-clinical’ PND symptoms at baseline had less improvement in their PND and PNA symptoms between baseline and week eight than those with ‘clinical’ symptoms at baseline. There was no difference identified between site or education for either EPDS or EPDS-3a (Tables 3 and 4).

## Discussion

The purpose of this study was to evaluate the Tresillian PNDA group therapy program. The results indicated that the program was effective in reducing symptoms of postnatal depression and anxiety and this improvement were still present at a four week ‘program reunion’ session. Overall, EPDS score reduced from an average of 14 at baseline to 10 at the end of the program. A four point change in EPDS is a clinically meaningful change (Matthey, 2004) and depicts improvements in symptoms from those that would be described as ‘clinical’ (i.e. EPDS above 13) to below clinical. The program was effective in reducing symptoms of postnatal depression and anxiety for many women including those with a previous history of mental illness symptoms and those with symptoms within the clinical range for PND. Those experiencing sub-clinical depression and anxiety symptoms did not have the same degree of positive change than those with symptoms within the clinical range. The addition of a sequential COS-P group had no effect in further reducing symptoms of depression or anxiety. There was difference between some groups in terms of the effect of the program on participant symptoms of depression.

These results add to the growing evidence base supporting the effectiveness of group psychotherapy in the postnatal period (Scope et al., 2013; Simhi et al., 2021). An Australian randomised controlled trial with a sample of postnatal women with diagnosed major depression found group psychotherapy (group interpersonal psychotherapy) to be superior to usual care in reducing depressive symptoms (Mulcahy et al., 2010). Likewise, two Canadian randomised controlled trials among a sample of perinatal women with diagnosed anxiety disorder (Green et al., 2020) or PND symptoms (Amani et al., 2021) found those participating in group psychotherapy (Cognitive behavioural group therapy) had reduced depression (Amani et al., 2021) and anxiety symptoms compared to a waitlisted control group (Green et al., 2020). In contrast to these studies, the current study is an evaluation of a clinical therapy group which includes a diverse population of women including those with and without a formal diagnosis of PND and/or PNA and those with and without a history of mental illness.

History of mental illness is a known risk factor for PND and PNA (McCarter-Spaulding & Shea, 2016). In the current study those with a history of mental illness on average started and finished with higher depression and anxiety symptoms than those without a history of mental illness but had a similar degree of improvement as those without a history of mental illness. This suggests that group therapy is effective for both groups. In group therapy not all group members have complete recovery (Mulcahy et al., 2010) and may require further alternative treatment options (e.g. individual psychotherapy, medication) (Reay et al., 2012). Another known risk factor for PND and PNA is lower socio-economic status (McCarter-Spaulding & Shea, 2016), based on the proxy measures of site and education captured in this study there was no difference in changes of symptoms of depression and anxiety by socio-economic status of the area. A caveated to this finding is the sample was highly educated, compared to the general population, a pattern consistent with recent studies that show women accessing tertiary referral child and family health service, like Tresillian, are more socio-economically advantaged (Dahlen et al., 2019).

Those participants who at the start of the program had sub-clinical depression symptoms (i.e. EPDS < 13) had a smaller degree of improvement compared to those with symptoms in the clinical range. For these participants it may be that participation in the group program prevented more severe symptoms, however a control group comparison is needed to prove this theory. Evidence for the group psychotherapy in the postnatal period as a prevention, rather than treatment, of PND and PNA is limited (Dennis & Dowswell, 2013). Available evdience suggests individual, rather than group interventions as most successful in prevention of PND (Dennis & Dowswell, 2013) and most prevenative interventions commenced in the prenatal, rather than postnatal period (McCarter-Spaulding & Shea, 2016). Therefore, further evidence is needed regarding prevention in general and specifically for group-based interventions commencing in the postnatal period.

Tresillian is a tertiary referral child and family health service in NSW Australia. Parents access these services often due to infant regulatory problems or concerns such as sleep, crying, or feeding problems (Dahlen et al., 2019; Priddis et al., 2018). Poor maternal mental health and maternal-infant relationship often co-occur with these regulatory problems (Olsen et al., 2019). The clinical presentation of women accessing these services often includes fatigue/exhaustion or anxiety (Priddis et al., 2018) and higher rates of PND or PNA are observable in women attending these services compared to the general population (Christl et al., 2013). This clinical context, along with the prevailing evidence to support targeting interventions to those women ‘at risk’ of PND (Dennis & Dowswell, 2013) and the findings of the present study all support offering the Tresillian PNDA group therapy program to those with both sub-clinical and clinical symptoms of PND and PNA.

The group attending the COS-P sequential to the Tresillian PNDA group therapy did not show any further improvement in depression and anxiety symptoms, compared to the group attending the Tresillian PNDA group therapy only. Other studies, examining COS-P as a stand-alone group intervention with similar cohorts of parents have identified that COS-P can reduce stress (Kohlhoff et al., 2016) and depression symptoms (Maxwell et al., 2021). While there is some evidence that the COS-P does reduce depression symptoms it is unclear which aspect of the program produces this effect, it could be, as examples, the act of being part of a group and/or the therapeutic relationship with the group facilitator (Maxwell et al., 2021). Hypothetically, if these examples are the aspects of the program producing this effect on depression symptoms, then the potency would be less for participants who had already received eight weeks of group-based therapy. Therefore, a larger sample size may have been needed to detect further reduction in depression symptoms. Nevertheless, considering the known link between PND/PNA and infant-mother attachment (Holt et al., 2021; Slomian et al., 2019) COS-P compliments the Tresillian PNDA group therapy by increasing a parent’s ability to observe, identify their infant’s needs and increased understanding of their infant’s behaviour (Kohlhoff et al., 2016; Powell et al., 2013).

Importantly, the act of being part of a group and/or the therapeutic relationship with the group facilitator are key to group therapy and quality and consistent participation is a critical factor for successful outcomes in group therapy (Yalom & Leszcz, 2005). Given this, the facilitator role in building a therapeutic alliance, facilitating a safe space and fostering ongoing participation can be critical to individual success in reducing PNDA symptoms (Scope et al., 2012). Therefore, it is not surprising that there was significant difference in depression symptoms between groups. This is an important consideration when assessing the efficacy and applicability of group therapy. 

Implications for practice and further research.

This study contributes to the body of evidence to support the use of group psychotherapy for women experiencing anxiety and depression in the postnatal period. The Tresillian PNDA group therapy was effective for a significant proportion of women who attended representing wide range of experiences (e.g. women with different age children, varied histories of mental illness and wide range of severity of symptoms at baseline). While this indicates that group psychotherapy works for a large group of women further research into who would benefit most from group psychotherapy and who would benefit from individual therapy is warranted. Likewise, not all groups shared the same improvements, understanding the features of successful group dynamics would be useful to future group programs. Future studies should also test the hypothesis of postnatal group psychotherapy as a prevention of depression and anxiety. The inclusion of the sequential attachment-based parenting program (COS-P) made no detectible difference in depression or anxiety symptoms in addition to those already achieved through the Tresillian PNDA group therapy. However, this does not negate the advantage of running the groups sequentially, with benefits for parent understanding of infant behaviour and understanding of attachment theory. Future studies should focus on tools used to identify changes in parenting observation and behaviour in response to their infant’s needs and behaviour.

### Study Strengths and Limitations

The strengths of this evaluative study include the design that measures effect of an intervention during typical clinical conditions (Furer et al., 2021) strengthening the ecological validity of the findings for community based parenting services. Due to the sample being taken during typical clinical conditions it was not homogenous. Which could be seen as limitation however, this heterogeneity represents the target population and therefore the results are potentially more applicable to the clinical context than if a restricted, more homogenous sample was used (Giraudeau et al., 2022).On the other hand, we were unable to collect data on those who did not consent to be part of the study to identity if the sample was representative of the eligible population. However, this sample is similar to those accessing tertiary referral child and family health service (e.g. Priddis et al., 2018). Based on available EPDS data collected prior by the organisation among the target group, the study was powered (80%) to detect a difference of 1.5 or more on the EPDS scale at the 5% significance level, by enrolling 58 or more participants at baseline. We ultimately enrolled 141 patients at baseline to be well positioned in terms of power to detect differences over time. Post-hoc observed power for the repeated measures analysis was 0.99, indicating adequate power was achieved.

Further limitations of this evaluation include loss to follow-up, the absence of a control group and unmeasured confounders for example, there was also no data collected on medication use and clinical diagnosis of depression or anxiety. Likewise, we did not identify in the data collected if the participants had received other individual psychotherapy treatment externally or within Tresillian. This evaluation included a large proportion of participants who were lost to follow-up (i.e. who missed data collection at time two and three), a dropout rate is expected in any group therapy programs but further analysis of reasons for drop out would be valuable in future evaluations. The included sample were highly educated and may represent a more socially advantaged demographic who access early parenting services more often (Dahlen et al., 2019). Additionally, certain demographic details such as ethnicity was not collected which has implication for the external validity. Research on the efficacy of group psychotherapy for PND/PNA amongst more socially disadvantaged or minority groups is warranted.

## Conclusion

Postnatal depression and anxiety cause significant distress for women with infants. Critically these conditions can compromise mothers’ mental and physical health and wellbeing and the infant’s development and health outcomes (Slomian et al., 2019). The Tresillian PNDA group therapy is effective in reducing both depression and anxiety in women with a previous history of mental illness symptoms and those with symptoms within the clinical range for PND. For women experiencing sub-clinical depression and anxiety symptoms in the group program may have facilitated prevention of more severe symptoms further research is needed to prove this theory.

## Electronic Supplementary Material

Below is the link to the electronic supplementary material.

Supplementary Material 1
