# Supplementary material for: Evaluation of Group Therapy Intervention for Anxiety and Depression in the Postnatal Period
Source: Matern Child Health J. 2025 Mar 26;29(4):537–48. doi: 10.1007/s10995-025-04076-9 (PMC12006229; doi:10.1007/s10995-025-04076-9)
Supplement: Supplementary file 1 — Supplementary Material 1 [file 10995_2025_4076_MOESM1_ESM.docx]

Appendix 1

Table 1 Comparison of participants included (n=85) and excluded (n=56) from final analysis

| **Variable** | **Included**  **(n=85)** | **Excluded**  **(n=56)** | **Missing** | **Statistic** |
| --- | --- | --- | --- | --- |
| **EPDS baseline score** |  |  | n=16 |  |
| Clinical | 56 (65.9%) | 22 (39.3%) |  | **χ^2^** (1, 125) = 2.10  *p* = .147 |
| Sub-clinical | 27 (31.8%) | 20 (35.7%) |  |  |
| **Group** |  |  | n=0 |  |
| PND | 65 (76.5%) | 27 (48.2%) |  | **χ^2^** (1, 141) = 10.67  ***p* = .001** |
| PND+COS-P | 20 (23.5%) | 29 (51.79%) |  |  |
| **Site** |  |  | n=0 |  |
| Site 1 lower socioeconomic area^ | 35 (41.2%) | 47 (83.9%) |  | **χ^2^** (1, 141) = 23.63  ***p* < .001** |
| Site 2 higher socioeconomic area | 50 (58.8%) | 9 (16.1%) |  |  |
| **Mother age** |  |  | n=13 |  |
| 20-24 | 1 (1.2%) | 0 |  | **χ^2^** (5, 128) = 9.17  *p* = .103 |
| 25-29 | 4 (4.7%) | 4 (7.1%) |  |  |
| 30-34 | 25 (29.4%) | 20 (35.7%) |  |  |
| 35-39 | 42 (49.4%) | 17 (53.6%) |  |  |
| 40-44 | 4 (4.7%) | 9 (16.1%) |  |  |
| 44+ | 1 (1.2%) | 1 (1.8%) |  |  |
| **Mother Highest education** |  |  | n=28 | **χ^2^** (5, 113) = 2.79  *p* = .733 |
| Secondary School | 2 (2.4%) | 0 |  |  |
| Higher Secondary School | 1 (1.2%) | 0 |  |  |
| Certificate or Diploma | 10 (11.8%) | 8 (14.3%) |  |  |
| Bachelor’s Degree | 34 (40%) | 16 (28.6%) |  |  |
| Post-Graduate Certificate or Diploma | 6 (7.1%) | 3 (5.4%) |  |  |
| Masters or Doctoral degree | 20 (23.5%) | 13 (23.2%) |  |  |
| **Number of Children** |  |  | n=28 |  |
| 1 | 54 (63.5%) | 28 (50%) |  | **χ^2^** (3, 113) = 1.20  *p* = .830 |
| 2 | 15 (17.6%) | 10 (17.9%) |  |  |
| 3 | 3 (3.5%) | 2 (3.6%) |  |  |
| 4 | 1 (1.2%) | 0 |  |  |
| **History of Mental illness** |  |  | n=34 |  |
| Yes (and not sure) | 47 (55.3%) | 28 (50%) |  | **χ^2^** (1, 107) = .005  *p* = .943 |
| No | 21 (24.7%) | 11 (19.6%) |  |  |
| **Youngest Child age** | n=80 | n=40 | n=21 |  |
| Mean (SD), range | 6.4 months (SD 3.5), 1.2-17 months | 7.8 months (SD 6.3), 1.0 – 36 months |  | *t* = 1.253 (21.2)  *p* = .22 |
| **Number of sessions attended** | n=54 (63.5%) | n=21 (37.5%) | n=66 |  |
| Mean, range | 7.2, 4-8 sessions | 6.5, 1-8 sessions |  | U = .489,  p = .658 |

Table 2 Repeated measures ANOVA: available case subgroup analysis

|  | **EPDS score (95% CI)** | | | | *Overall statistic* |
| --- | --- | --- | --- | --- | --- |
|  | Baseline | Post-group (week 8) | | 4-week follow up (week 12) |  |
| All (n = 53) | 14.19  (13.18 – 15.20) | 9.77  (8.72 – 10.83) | | 9.72  (8.41 – 11.03) | *F* = 34.24 (1.73, 89.89), *p* < .001 |
| *Contrast statistic* | *F* = 50.74(1, 52), *p*  < .001 | | *F* = .13(1, 52), *p* = .908 | |  |
|  | **EPDS-3a score (95% CI)** | | | |  |
|  | Baseline | Post-group (week 8) | | 4-week follow up (week 12) |  |
| All (n = 53) | 5.83  (5.38 – 6.28) | 4.37  (3.91 – 4.84) | | 4.34  (8.81 – 4.88) | *F* = 24.87(2, 104),  *p* < .001 |
| *Contrast statistic* | *F* = 34.81(1, 52), *p* < .001 | | *F* = .03(1, 52), *p* = .865 | |  |

Table 3 Factorial mixed ANOVA EPDS by group – between group effects parameter estimates

| **Group ID** | **Pre** | **Post** | **Reunion** |
| --- | --- | --- | --- |
| 14 | 0 (reference group) | 0 (reference group) | 0 (reference group) |
| 1 | **-4.393 (-8.235 - -.550)^a^** | -.464 (-4.682 - 3.754) | -.953 (-5.124 - 3.219) |
| 3 | 1.528 (-2.080 - 5.135) | -.639 (-4.599 - 3.321) | -1.320 (-5.237 - 2.596) |
| 4 | -2.403 (-6.011 - 1.204) | -1.194 (-5.155 - 2.766) | -.887 (-4.803 - 3.030) |
| 5 | -2.083 (-6.093 - 1.926) | 2.083 (-2.318 - 6.485) | .645 (-3.708 - 4.998) |
| 6 | 1.607 (-2.235 - 5.450) | 1.393 (-2.825 - 5.611) | .297 (-3.874 - 4.469) |
| 7 | 2.639 (-.969 - 6.246) | -2.528 (-6.488 - 1.433) | -2.442 (-6.359 - 1.474) |
| 8 | 1.893 (-1.950 - 5.735) | .387 (-3.831 - 4.605) | .257 (-3.914 - 4.429) |
| 9 | .179 (-3.664 - 4.021) | **-4.036 (-8.254 - .182)^b^** | -3.367 (-7.538 - .805) |
| 12 | -.083 (-4.093 - 3.926) | -1.117 (-5.518 - 3.285) | .235 (-4.118 - 4.588) |
| 13 | -.950 (-4.472 - 2.572) | -1.210 (-5.076 - 2.656) | .694 (-3.129 - 4.517) |

**^a^*p*=.026; ^b^*p*=.060;** all other p-values >.1
